# Integrated fMRI Preprocessing Framework Using Extended Kalman Filter for Estimation of Slice-Wise Motion

**DOI:** 10.3389/fnins.2018.00268

**Published:** 2018-04-26

**Authors:** Basile Pinsard, Arnaud Boutin, Julien Doyon, Habib Benali

**Affiliations:** ^1^Unité de Neuroimagerie Fonctionelle, Centre de Recherche de l'Institut Universitaire de Gériatrie de Montréal, Montreal, QC, Canada; ^2^UMR7371 Laboratoire d'Imagerie Biomédicale, Paris, France; ^3^Sorbonne Universités, Paris, France; ^4^Montreal Neurological Institute, McGill University, Montreal, QC, Canada; ^5^PERFORM Center, Concordia University, Montreal, QC, Canada

**Keywords:** fMRI, motion correction, distortion correction, denoising, BOLD, visualization

## Abstract

Functional MRI acquisition is sensitive to subjects' motion that cannot be fully constrained. Therefore, signal corrections have to be applied a posteriori in order to mitigate the complex interactions between changing tissue localization and magnetic fields, gradients and readouts. To circumvent current preprocessing strategies limitations, we developed an integrated method that correct motion and spatial low-frequency intensity fluctuations at the level of each slice in order to better fit the acquisition processes. The registration of single or multiple simultaneously acquired slices is achieved online by an Iterated Extended Kalman Filter, favoring the robust estimation of continuous motion, while an intensity bias field is non-parametrically fitted. The proposed extraction of gray-matter BOLD activity from the acquisition space to an anatomical group template space, taking into account distortions, better preserves fine-scale patterns of activity. Importantly, the proposed unified framework generalizes to high-resolution multi-slice techniques. When tested on simulated and real data the latter shows a reduction of motion explained variance and signal variability when compared to the conventional preprocessing approach. These improvements provide more stable patterns of activity, facilitating investigation of cerebral information representation in healthy and/or clinical populations where motion is known to impact fine-scale data.

## Introduction

Functional MRI (fMRI) with echo-planar-imaging (EPI) is widely used in neuroscience research to indirectly measure brain activity through the brain-oxygen-level-dependent (BOLD) signal. Yet subject motion during fMRI scanning causes important signal changes unrelated to the hemodynamic response of interest (for reviews see Zaitsev et al., 2015, 2017) resulting mainly from variations in partial voxelwise tissue volumes with different relaxation times. Motion induced detrimental contrast and intensity changes also originate by the spin-history effects resulting from disruption of the *T*_1_ steady state (Muresan et al., 2002) and loss of magnetization between excitation and readout. In addition, EPI suffers from gross non-linear distortions and signal losses due to main static field (B0) in-homogeneity (Jezzard and Balaban, 1995) caused by interfaces between air and subjects' tissues or bones, which when moved in the B0 field modulate these artifacts. The latter artifacts as well as physiological noise are even more evident at high fields (e.g., 3 or 7 Tesla) (Triantafyllou et al., 2005; Bollmann et al., 2017) mitigating the benefit in signal sensitivity. Related to these issues is the fact that numerous innovations in pulse acquisition sequences, and simultaneous multi-slice imaging (SMS) (Feinberg and Setsompop, 2013) in particular, have rapidly been adopted in leading research initiatives such as the Human Connectome Project (HCP) (Uğurbil et al., 2013; Van Essen et al., 2013). This type of functional sequence enables simultaneous imaging of multiple 2D slices which, combined with in-plane acceleration, allows high spatial resolution and/or sampling rate. However, the increase of in-plane resolution extends the readout echo train and concerns have thus been raised about a heightened sensitivity to motion and respiration induced B0 in-homogeneity fluctuations under those conditions.

Such entangled MRI-related phenomena, magnified by novel acquisition techniques, generate important non-linear signal perturbations. Differences in motion amplitude and occurrence across groups of subjects can then result in strong spurious effects in subsequent statistical analyses (Power et al., 2012). This is even more manifest in participants for which motion control is difficult, such as in clinical (Haller et al., 2014), older (Mowinckel et al., 2012) and pediatric (Power et al., 2012; Satterthwaite et al., 2012) populations, or when responses to a particular task introduces subject's motion during scanning.

Despite aforementioned limitations, improvements in measurements through innovations in MRI hardware and acquisition sequences have helped the neuroimaging field to constantly move its interest to finer characteristic of brain activity using complex experimental designs and elaborated analysis techniques. Notably, multivariate pattern analysis (MVPA) has received major attention in recent years (Pereira et al., 2009; Haxby, 2012), allowing novel hypotheses to be tested. Such techniques look for fine-scaled spatio-temporal activity patterns, but conventional preprocessing pipelines perform successive interpolations, resulting in low-pass spatial filtering. While further spatial smoothing is voluntarily applied to increase the robustness of classically investigated broad activity signal, it also averages out high frequency information across neighboring but distinct neuronal populations and structures such as the opposite cortical sulci banks.

In order to address such spatial alignment issues, two main preprocessing approaches have been proposed. First, motion correction can be performed prospectively by adjusting slices position during scanning in order to stabilize the anatomical coverage using either MRI data of preceding volume, interleaved navigators (White et al., 2010) or external motion tracking devices. Second, due to technical difficulties (i.e., the need to use physical trackers and adapt pulse sequence) in implementing prospective methods, most researchers have been using a retrospective approach, which consists in estimating motion from fMRI volumes that are then realigned. However, the available implementations of both techniques perform 3D volume registrations, thus dismissing the fact that sequential acquisition of EPI slices does not guarantee that motion occurs solely between volumes. As intra-volume motion can be frequent, the single registration estimate of each 3D volume averages the different spatial positions of slices, depending on their information content, hence causing local misregistrations. In addition, both scanner noise and fMRI resolution also limit the stability of these successive estimates, resulting in biologically unrealistic detected motion due to noisy registration. This, in turn, leads the corrected data, parametrized by these estimates, to contain long-range spurious covariance extending beyond motion-corrupted slices (Satterthwaite et al., 2013). To account for motion induced signal changes, the preprocessing often regresses out of the BOLD signal, the estimated movement parameters, sometimes differentiated and squared (Satterthwaite et al., 2013; Yan et al., 2013). While these noisy and inaccurate volume-wise motion parameters can mitigate spurious signal changes introduced by the resulting imperfect realignment, they cannot fully account for non-linearity of magnetic field and gradient changes entangled with physiological processes and scanner noise (Power et al., 2014).

Such limitations in volume-wise registration motivated the development of slice-to-volume (S2V) motion correction methods (Kim et al., 1999; Yeo et al., 2006, 2008; Ferrante and Paragios, 2017) for a review, which perform individual registration of each slice to a reference 3D volume. To circumvent the scarcer information content of a single EPI slice these methods implement a top-down registration scheme (Ferrazzi et al., 2014), additional stabilization (Park et al., 2004), regularized iterative global optimization (Gholipour et al., 2010; Seshamani et al., 2014) or an outlier detection (Marami et al., 2016) approach. By better fitting the acquisition process, S2V approaches provide improved signal, notably in applications prone to large motion such as fetal imaging. Yet these softwares are not shared among members of the scientific community and are thus not widely used in fMRI research. To correct motion induced signal changes, linear data-driven methods (Perlbarg et al., 2007; Griffanti et al., 2014) have then been applied, first to resting state and then to task-related data, trying to remove volume-wise structured noise that remains after motion correction. However transient slice-specific artifacts are already non-linearly propagated to neighboring slices through interpolation, hence complicating their removal. Thus, as S2V improves registration, EPI-based fMRI would also benefit from slice-specific signal corrections in acquisition space prior to any volumetric processing.

Therefore, there is a need for fMRI correction strategies that better take into account the MRI acquisition specificities and adapt to novel high-resolution imaging techniques such as SMS, which generate larger datasets and thus require more efficient preprocessing, analysis and data storage (Glasser et al., 2013). To fulfill such a need, we propose here an SMS compatible integrated retrospective method for slice-wise motion, distortion and signal correction as well as extraction of gray matter BOLD fine-scale patterns. The main contribution of this development is a preprocessing framework build around the specificities of widely used sliced acquisition, in which existing and novel processing methods are combined in a sensible way, and could integrate other slice-specific processing in further work. A thorough evaluation of the method is performed on both simulated and real data.

## Methods

### Motion estimation

**Figure 1:**
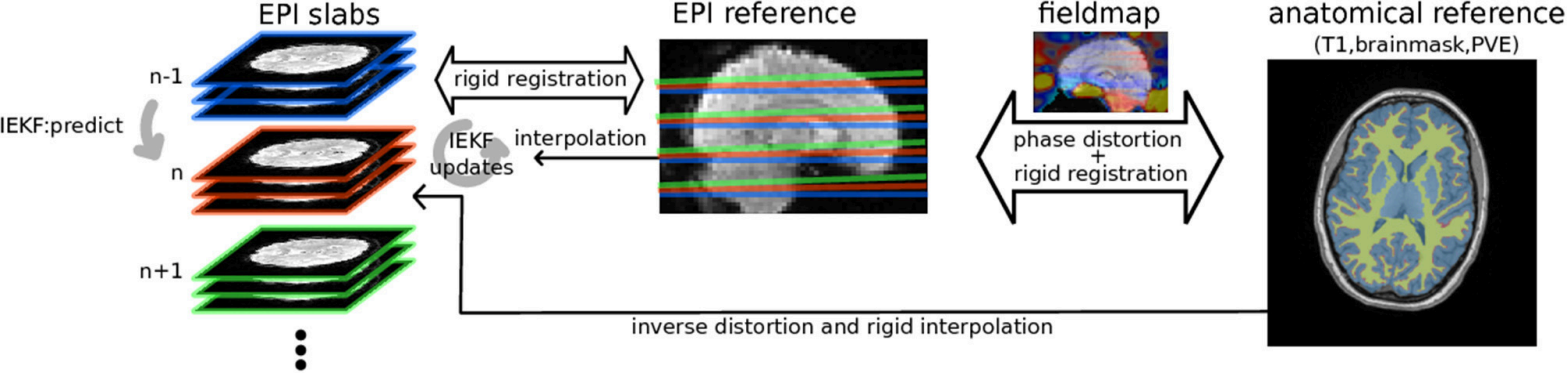
Diagram of the algorithm: EPI groups of slices are sequentially rigidly registered by IEKF to the EPI reference initialized by previously estimated position, while concurrently removing an intensity bias using prior information (mask, partial volume effect map) from anatomical segmented anatomical scan while taking into account distortions estimated from B0 fieldmap.

Starting from an initial registration of the first EPI full volume to T1 reference, the motion estimation algorithm then iterates through groups of slices or single slices of SMS or conventional acquisition scheme respectively as illustrated in Figure 1. To model continuously the subjects' head changing position, we chose an Iterated Extended Kalman Filter (IEKF); a derivation of standard Kalman Filter that takes into account the non-linearity of MR signal changes described above through local derivation and iterative optimization. The IEKF, previously used in navigator-based prospective motion correction for MRI anatomical scan (White et al., 2010), allows to robustly track hidden states of 3D translation and rotation, a six parameters vector *x*_*t*_, *t* ∈ [0..*T*] from observation. For each group of slices acquired at time *t* the algorithm makes a prediction *x*_*t*_ of the state and *P* of the covariance of this prediction error:

(1) $$\begin{array}{l} x_{t|t-1}^{0}=Ax_{t-1|t-1} \end{array}$$

(2) $$\begin{array}{l} P_{t|t-1}^{0}=P_{t-1|t-1}+Q \end{array}$$

*A* = *I*_6_ the transition matrix is defined as identity, as it is the best estimate for the head position when the next slice is acquired. *P* can be initialized as a diagonal matrix and *Q* is added to the covariance of the previous slice to predict the error accounting for the possible change in position during the time δ*t* separating the acquisition of the two slices.

This prediction is recursively updated using the acquired data *y* by iterating for *i* = 1..*N* until convergence or maximum number of iterations is attained:

(3) $$\begin{array}{l} K_{t}^{i}=P_{t|t-1}{H^{i}}^{T}{\left[H^{i}P_{t|t-1}{H^{i}}^{T}+R\right]}^{-1} \end{array}$$

(4) $$\begin{array}{l} x_{t|t}^{i+1}=x_{t|t-1}^{i}-K_{t}^{i}\left[y-h\left(x_{t|t}^{i}\right)\right] \end{array}$$

with *K* the Kalman gain matrix, *H* being the Jacobian based on finite difference of the measurement function *h* that samples the reference, and *R* the observation covariance, approximated by a diagonal covariance matrix with observation variance allowing weighting of the voxels' influence on registration depending on their reliability.

After this iterative optimization, state covariance is then updated:

(5) $$\begin{array}{l} P_{t|t}=\left[I-K_{t}^{N}H^{N}\right]P_{t|t-1} \end{array}$$

This iterative updating, similar to a gradient-descent or Gauss-Newton algorithm, allow non-linear optimization which initialized to the most recently estimated position, is likely to converge to a global optimal solution. The updating is performed with partial observation, thus the cost function and its Jacobian are only estimated for the part of the brain imaged in the group of slices, by interpolating the brain mask from the anatomical reference space.

### Intensity bias correction

Apart from the misalignment, the motion induces multiple contrast and intensity modulations of the signal (Zaitsev et al., 2017), notably due to non-linear changes of the static field in-homogeneity and the loss of slice excitation or magnetization steady-state. Independent of the changes in subjects' position, the static field and gradients can also show some non-linearity and instability (Bollmann et al., 2017) respectively due to gradient coil and passive shimming heatings, that causes contrast and intensity changes (Foerster et al., 2005; El-Sharkawy et al., 2006). As these intensity changes are specific to individual slices, it is thought that the best procedure is to correct raw acquired slices before any interpolation is performed with the rationale of avoiding to mix slice signals and artifacts through interpolations which might then prove difficult to separate.

As with other bias correction methods (Seshamani et al., 2014), we choose to model intensity changes using the assumption of spatially smooth multiplicative effect on the measured signal. This approach thus do not specifically model any of the acquisition phenomena describe above, but rather aims at removing spatial low-frequency changes in the signal that are unrelated to the BOLD effect of interest, similar to correction based on signal decomposition techniques (Perlbarg et al., 2007; Griffanti et al., 2014).

In that goal, the update step of the IEKF is modified to incorporate a bias correction term:

(6) $$\begin{array}{l} x_{t|t}^{i+1}=x_{t|t-1}^{i}-K_{t}^{i}\left[\frac{y}{b}-h\left(x_{t|t}^{i}\right)\right] \end{array}$$

where *b* is the bias field estimated, at each iteration of IEKF, for each 2D slice separately, using the following non-parametric measure:

(7) $$\begin{array}{l} b\left(x_{i}\right)=\frac{\sum_{j\in \Omega }\phi_{\sigma_{b}}\left(d_{ij}\right)w\left(j\right)y\left(j\right)}{\sum_{j\in \Omega }\phi_{\sigma_{b}}\left(d_{ij}\right)w\left(j\right)h\left(x_{t|t}^{i},j\right)} \end{array}$$

where *d*_*ij*_ is the euclidean distance between voxels *i* and *j*, Ω constitutes the set of voxels in the slices containing voxel *i*, and ϕ is a Gaussian kernel of standard deviation σ_*b*_ that control the spatial frequency to be removed and w(j) is a weight parameter. This equation computes a weighted estimate of the spatial low-frequency intensity differences between the slab being processed and the reference. White matter partial volume map is used as weight *w*, as it is expected that intensity changes are limitedly related to neural activity, and is widely used for confound regression. Gray matter bias values are thus extrapolated from white-matter through Gaussian kernel allowing intensity correction without removal of signal changes of interest. Removing the low spatial frequencies of the differences between the slice being processed and the reference is expected to improve the similarity measure used in the IEKF registration.

### Implementation

The method was developed in python using the nipy package, and integrated in the nipype pipelining environment, both under open-source licence, and will thus be made available to the community. A pseudo-code is provided in appendix A.1 (Supplementary Material) to give an algorithmic view of the method.

#### Transformation and distortion correction

As mentioned above, motion estimation and bias correction steps require anatomical priors, namely the brain mask and the white matter partial volume map. As EPI shows localized gross distortion due to static magnetic field (B0) in-homogeneity, a linear mapping of undistorted anatomical priors cannot match the data being preprocessed. Distortions were thus applied to anatomical information when mapping these priors to EPI space, by applying a shift in the phase encoding direction as estimated from a mapping of the B0 field. The latter could be estimated either from a dual-echo sequence (Jezzard and Balaban, 1995; Hutton et al., 2002) acquired separately or an fMRI acquisition with reversed phase encoding paired to the one being processed (Andersson et al., 2003).

#### Interpolation and data storage

Once slabs composing each volume are successively registered and their signal is corrected in slice space, one still needs to extract timecourses of the BOLD signal at stable anatomical location of interest for studying brain activity. With individual slice registration, motion estimation of a single volume results in slices with varying position relative to anatomical space, in which voxels are shifted in phase encoding direction as estimated from the fieldmap. Hence, the functional voxels are located on an irregular grid in the anatomical space which poses a problem of scattered data interpolation. We solved this problem by implementing a Nadaraya–Watson Gaussian kernel regression (Nadaraya, 1964; Watson, 1964; Bierens, 1996), which for each anatomically defined gray-matter location *v*_*k*_ computed the average of neighboring scattered EPI voxels *x*_*i*_ intensity corrected by bias *b*(*x*_*i*_) and weighted by the distance *d*(*v*_*k*_, *x*_*i*_) transformed by Gaussian kernel ϕ.

(8) $$\begin{array}{l} I\left(v_{k},t\right)=\frac{\sum_{i}\phi_{\sigma_{rbf}}\left(d\left(v_{k},x_{i}\right)\right)y\left(x_{i},t\right)/b\left(x_{i},t\right)}{\sum_{i}\phi_{\sigma_{rbf}}\left(d\left(v_{k},x_{i}\right)\right)} \end{array}$$

In a previous implementation of volumetric to surface data interpolation (Operto et al., 2008) a kernel with anatomical constraints was used to avoid inclusion of white matter, cerebrospinal fluid or opposite sulci banks in interpolation of neocortical signal. To the same end, we allowed the use of an anisotropic Gaussian kernel with restricted extent in the direction normal to the cortical surface, complying with anatomical topology. Therefore, the subcortical grayordinates were resampled using a Gaussian kernel with 1.5 mm standard deviation, while the cortical grayordinates were resampled using a anisotropic Gaussian kernel with 1.5 and 0.5 mm standard deviation respectively for the directions tangent and perpendicular to the cortex.

As with the Human Connectome Project grayordinates (Glasser et al., 2013), our pipeline warps anatomical templates to individual subject space, including surface registered by spherical alignment (Dale et al., 1999; Fischl et al., 1999a,b), and volumetric ROIS coordinates through non-linear diffeomorphic warping (Avants et al., 2008). Therefore, the single interpolation described above directly provides signal anatomically aligned between individuals, allowing group-level analysis and visualization without further interpolation.

As the algorithm applies correction online, loading raw DICOM files, it would allow real-time analysis of the data, if computational performance and processing delay meet the application requirements. Online storage of interpolated signal also reduces memory usage, enabling processing of long and high temporal or spatial resolution acquisitions resulting from accelerated or multiband imaging. The chosen storage format uses HDF5 standard, providing fast and flexibly accessible storage of large dataset on disk. Altogether, this overcome the limitation of current methods which, depending on implementation, requires loading of the whole acquisition data in memory.

### Evaluation

#### Datasets

The choice of the datasets was motivated by the fact that we needed to test the method on a diversity of protocols including different experimental paradigms and type of sequences. Thus we used:

The Healthy Brain Network Serial Scanning Initiative (HBN-SSI) dataset (O'Connor et al., 2017), in which subjects were scanned in a series of 14 sessions aiming at test-retest reliability assessments. This dataset, acquired on a Siemens 1.5T Avanto scanner with 32-channel coil, includes resting-state and tasks related acquisitions with a multiband sequence (TR = 1,450 ms, TE = 40 ms, FA = 55, MB_factor = 3 × 18 = 54 slices interleaved ascending, res = 2.46 × 2.46 × 2.5mm), a dual-echo fieldmap, as well as an T1 MEMPRAGE (1 mm iso, FA = 7, TR = 2,730 ms, TE = 1.64 ms, GRAPPA = 2), a root-mean-square (RMS) was computed across echoes, the readers can report to the published data note (O'Connor et al., 2017) for the full description of this dataset.

A local protocol, acquired on a Siemens 3T Tim Trio B17, that included a motor sequence execution task designed for MVPA, which was performed in two separated scans for each of the 25 subjects, in a single-band acquisition (using 12-channel coil, TR = 2,160 ms, TE = 30 ms, FA = 90, 40 slices sequential ascending, res = 3.44 × 3.44 × 3.3 mm, GRAPPA = 2), to test the algorithm with both single-band scan sequence and pattern analysis. After the task was completed, a short acquisition with similar parameters but with reversed phase was acquired, for estimation of inhomogeneity induced distortions. In another session, a T1 MEMPRAGE (1 mm iso, TR = 2,530 ms, TE = 1.64, 3.6, 5.36, 7.22 ms, FA = 7, GRAPPA = 2) was acquired using a 32-channel, and the echoes were combined by computing the RMS. The task required the subjects to perform four different 5-keypress sequences with their left hand fingers excluding the thumb, each of the four sequences being repeated 5 times in 8 practice blocks, each scan including 32 pseudo-random ordered blocks. Each subject performed the task twice in two separate scans. This study protocol was approved by the Research Ethics Board of the “Regroupement en Neuroimagerie du Québec” (RNQ). All subjects provided written informed consent and received financial compensation for their participation.

#### Simulations

To assess the accuracy of our method, we first simulated fMRI data by taking the first volume of the fMRI sequences, and by introducing parametrized slice-wise motion and by resampling slices in this volume. The generated motion included both slow and rapid acceleration to assess the different types of motion that subjects head can show in the scanner. At each slice, the slow and fast accelerations had respectively a 10 and 5% chance to be changed, being randomly drawn from a logistic distribution with a mean biased toward the original position. This bias, reflecting the restriction of movements that the scanner and the coil impose to the head, avoided the head to move out of the field-of-view. With probability increasing with velocity, the fast acceleration was reset to zero and speed divided, progressively stopping the movement. The exact procedure for movement generation is provided in python code in appendix A.2 (Supplementary Material). We further introduced a smooth multiplicative spatial bias generated from a polynomial basis of degree 3 with random coefficients and we finally added noise drawn from a Rician distribution (Henkelman, 1985).

The simulations were based on the local protocol dataset, the first volume of the 2 scans of each of the 25 subjects set as input, yielding 50 simulations with different motion and bias field parameters.

#### Comparison to conventional pipeline

To compare with more conventional pipeline, we processed the same datasets using MCFLIRT (FSL) motion correction (Jenkinson et al., 2002), Topup (Andersson et al., 2003) or fieldmap based distortion correction (Hutton et al., 2002) depending on fieldmap or inverted-phase EPI availability. Then we extracted signals of interest in gray matter using volume to surface ribbon-constrained interpolation of HCP workbench tools (Glasser et al., 2013).

#### Signal characteristics

To evaluate the benefits of the novel estimation of motion we compared the statistical dependence between the estimated motion and resampled timeseries to that of the conventional pipeline. We thus computed the absolute derivative of the signal δ_∣*y*∣_(*t*) = ∣*y*(*t*) − *y*(*t* − 1)∣ from which we regressed the delta-root-mean-square (DRMS) (Power et al., 2012) of the motion: ${DRMS}_{x}\left(t\right)=\sqrt{\overset{6}{\underset{p=1}{\sum }}{\left(x\left(t,p\right)-x\left(t-1,p\right)\right)}^{2}}$, for *t* ∈ [1, *T* − 1]. An absolute value was taken as the motion-related intensity changes can have inverted signs across scans (Power et al., 2012).

As BOLD signal is functionally tied to the hemodynamic response function (HRF), it should exhibit characteristic temporal smoothness, despite underlying broadband neuronal activity. However, as noise has less limited frequency range, its variance added to the signal of interest can reduce this low frequency structure. To evaluate the motion and related signal intensity corrections, we measured smoothness of the signal as the variance of the derivative of the signal divided by the variance of the signal itself $\frac{\sigma \left(\delta_{y}\right)}{\sigma y}$. Slowly fluctuating signal should exhibit low derivative variance as compared to the signal variance, so the smallest this quantity the smoothest the timeseries.

## Results

### Simulation

**Figure 2:**
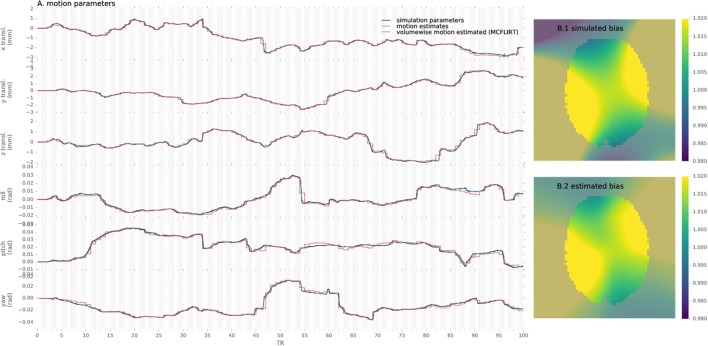
Example of motion parameters and bias simulated and estimated by the proposed algorithm and conventional volume-wise method.

To assert that the algorithm is able to reliably track the subjects' head motion, we processed the simulated data and measured the error in motion estimate compared to the simulated parameters for the proposed method and conventional preprocessing. An example of motion estimated with both methods as compared to simulated motion is provided in Figure 2.

Similarly, simulated and estimated biases are shown in Figure 2. As the bias is estimated only from the voxels including white matter, values in other voxels are extrapolated through the Gaussian kernel and thus error increases with distance from the white-matter, notably in gray-matter or outside of the brain mask.

**Figure 3:**
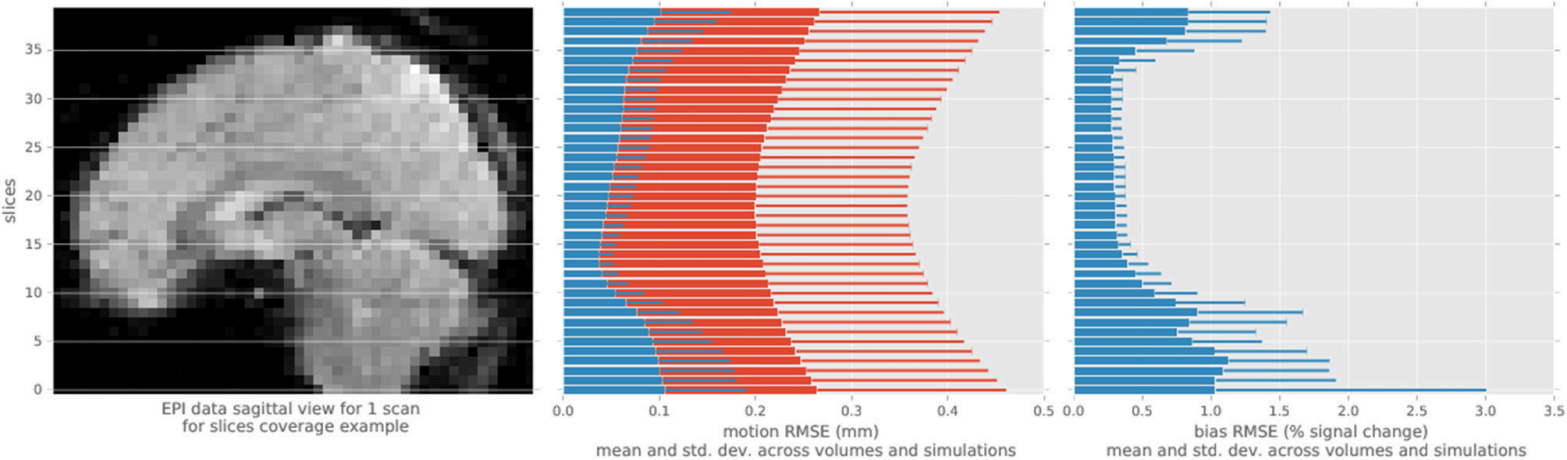
Root mean square error statistics of motion and bias estimates per slice across simulations, for the single-slice acquisition simulations, using the proposed algorithm (blue) and MCFLIRT (red). The top and bottom slices show higher errors in motion slicewise estimates and for conventional volume registration, as well as in bias estimation.

We evaluated the root-mean-squared error (RMSE) between the estimated and simulated motion and intensity bias in brain mask (Figure 3) in all single-slice sequence simulations. Rotational parameters were converted to translation, using a 100 mm radius, prior to computing the RMSE to obtain a single error measure for each slice simulated. The consistent pattern of error magnitude clearly exhibits the dependence of algorithm accuracy on slicewise available information for both motion and bias. With conventional preprocessing, the accuracy of volume-based registration similarly varies when assessed at the slice level, being also driven by the richer information of central slices. Slices at the top and bottom of the brain contains lower number of voxels covering the brain than middle slices, decreasing the accuracy of the registration process, and the number of voxel containing white matter follow the same pattern increasing the bias estimation error.

### Real dataset

We applied the algorithm to the real datasets, and extracted examples of the different estimated parameters to illustrate the results, including the bias map for a single slice in Figure S2.

To illustrate fieldmap-based distortion correction, we interpolated corrected EPI data to volumetric anatomical space in Figure S1 with T1 segmented white-matter boundary overlaid.

#### Quality assessment

**Figure 4:**
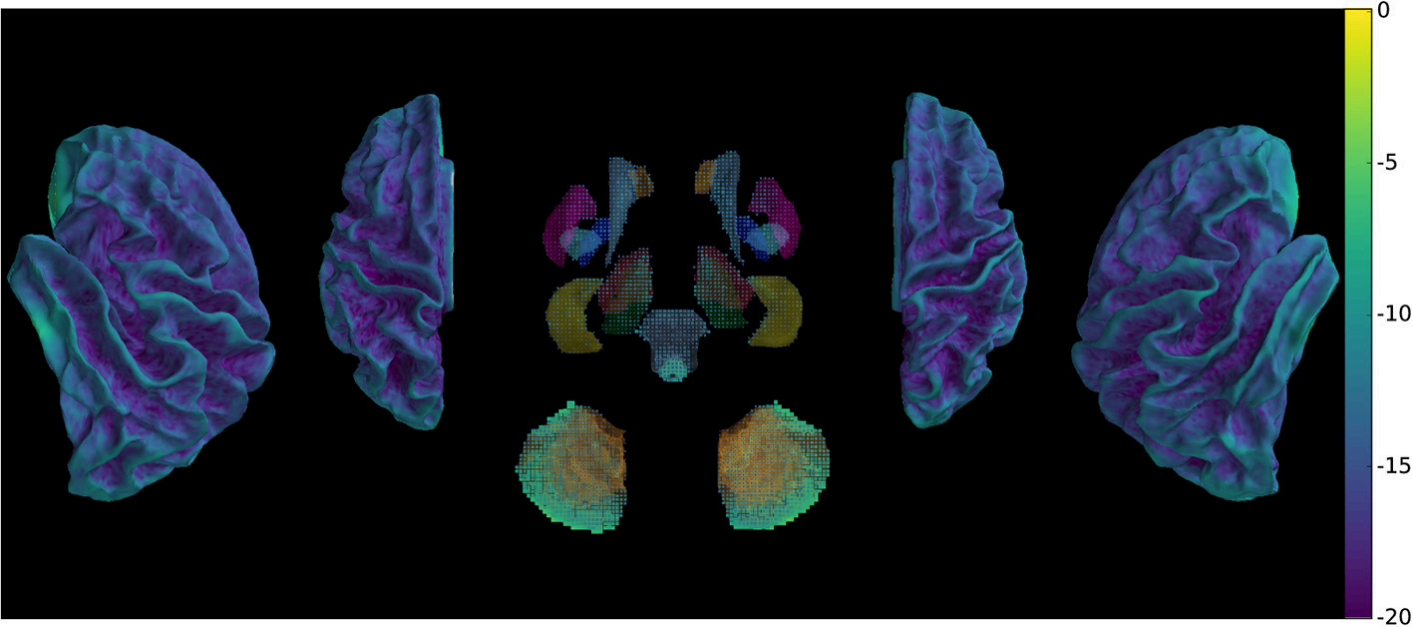
Comparison of betas of BOLD signal regressed with motion parameters (DRMS) using between-preprocessing paired *t*-test across subjects and scans, showing global decrease of motion explained variance.

To assess the preprocessed signal quality, we compared the variance that can be explained by the motion parameters in a regression model between the proposed method and conventional preprocessing. The betas were submitted to pairwise comparisons between the two methods across subjects and scans to generate a map (Figure 4), which shows differential reduction of motion explained variance of the signal depending on anatomy.

**Figure 5:**
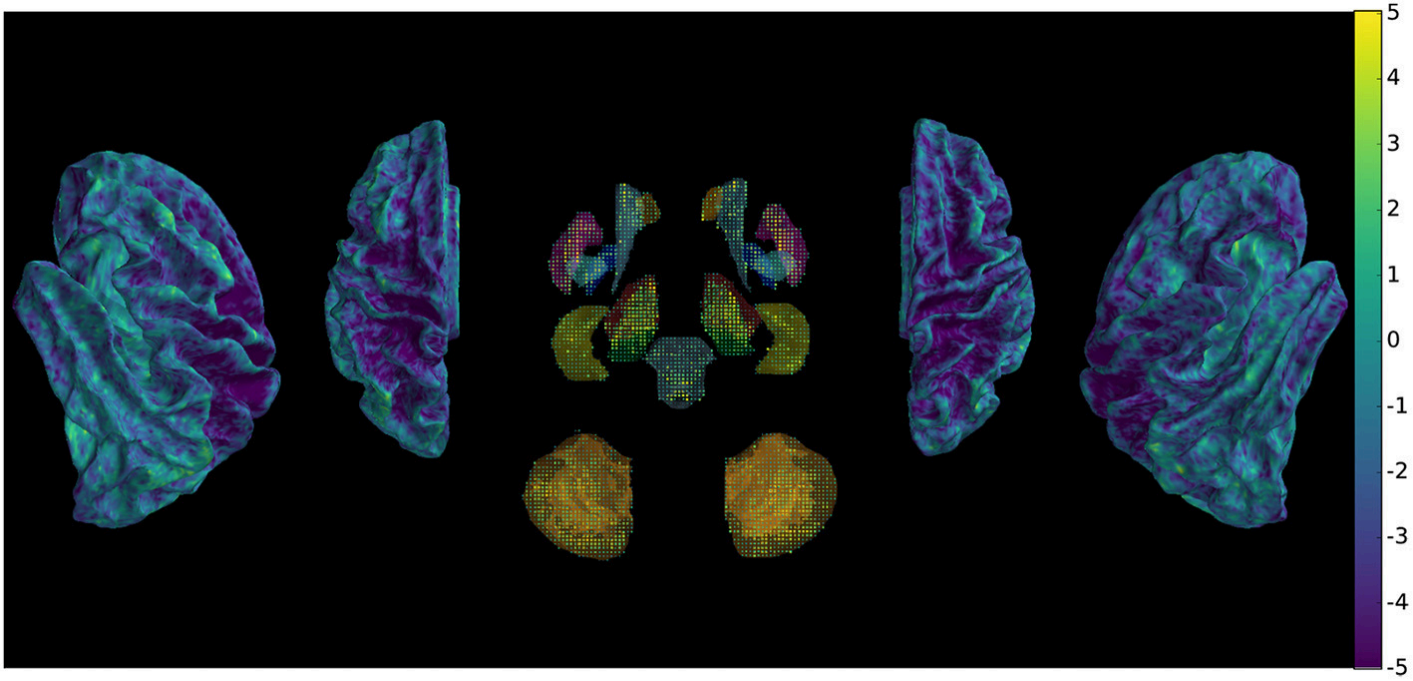
Comparison of signal variability between new and conventional preprocessing using a paired *t*-test across subjects and scans, showing overall decrease of rapid non-physiologically plausible signal changes.

The metric evaluating the variability of the signal was submitted to pairwise comparisons between the two methods across subjects and scans, which shows (Figure 5) widespread decrease of variability with few exceptions.

The difference in stability of the signal can result from the combination of different motion, distortion and intensity corrections.

#### Analysis results

**Figure 6:**
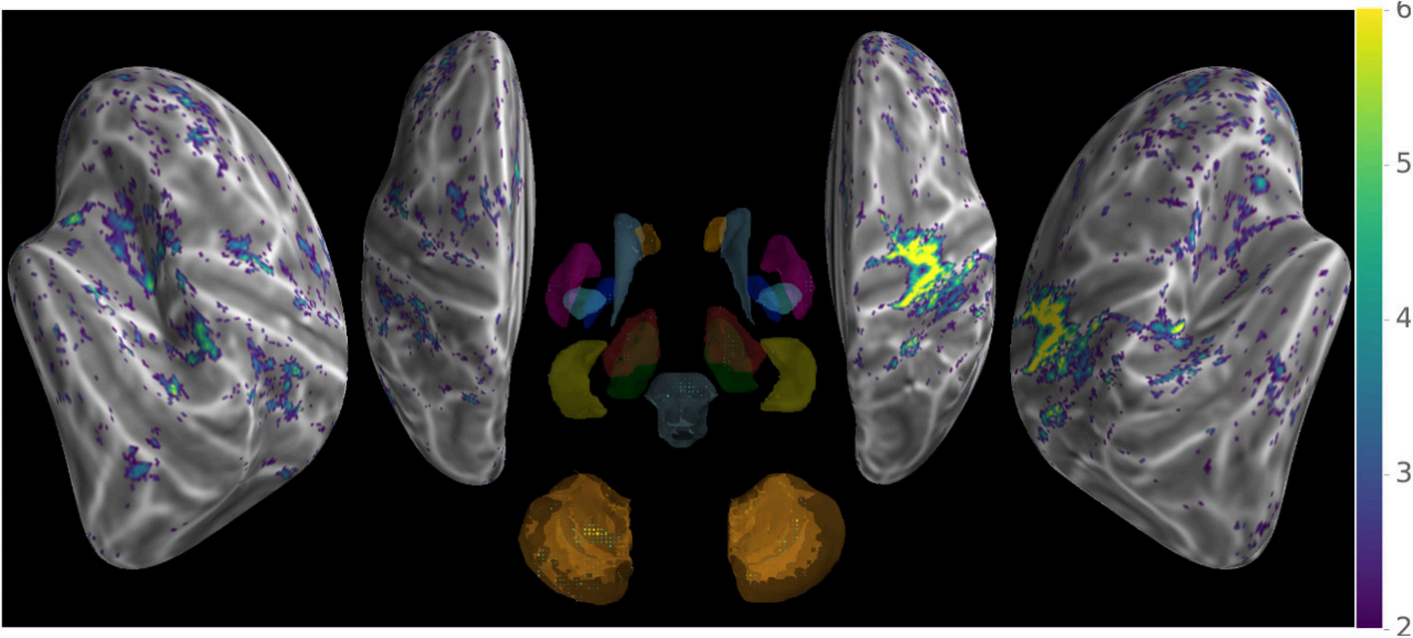
Wholebrain exploded view displaying a *T*-value map of an activation for a single block of about 5 s. during left-hand finger motor sequence production, showing activity in contralateral primary sensorimotor cortex and the anterior lobe of ipsilateral cerebellum.

Our method provides a sparse BOLD signal because of extraction of gray matter signals only. We therefore designed a 3D viewer to allow an exploded view of the whole brain results in a single image by representing the cortical surfaces, optionally inflated, of both hemispheres as well as subcortical structures represented as voxel cloud enclosed in transparent boundary surfaces colored by freesurfer atlas colors. The colormap represent the values of the displayed data overlaid on curvature grayscale values (as illustrated in Figure 6 with *t*-values), the sizes of the subcortical voxels also vary with these values to allow viewing of significant clusters.

## Discussion

Correcting motion in low-resolution noisy MRI data, such as EPI fMRI acquisition, has been a challenging problem since the introduction of functional imaging, even with the simpler rigid constraint inherent to brain imaging. Existing algorithms, required to be robust, achieve this correction in a sub-optimal way by notably performing volume-wise correction of separately acquired slices thus not taking into account the imaging process. While advances in scanner hardware and acquisition sequence development achieve higher resolution, opening finer investigation of physiological processes, these simpler methods are limiting their accuracy. In the meantime, functional imaging uses acquisition techniques prone to artifacts, including distortion and signal changes, these being amplified with novel hardware and the use of higher resolution.

Even small estimated motions have been found to cause spurious intensity changes in preprocessed signals (Power et al., 2012), although their interpolation is parametrized by the same motion estimates.

To address these limitations we extended early work on slice-to-volume motion correction (Kim et al., 1999; Yeo et al., 2006, 2008), with the method presented here, which concurrently corrects for EPI distortions and spurious intensity changes. To improve robustness of the registrations of sequentially acquired single slices or SMS slice-groups to a high resolution anatomical image, we modeled motion in a state-space and applied online Kalman filtering similarly to Marami et al. (2016). The distortion estimated from a fieldmap was used during the registration of slices to improve correspondence of EPI data to the non-distorted anatomical reference. EPI signal is corrupted by spatial low-frequency intensity changes due to magnetic fluctuations induced by scanner instabilities (Foerster et al., 2005; El-Sharkawy et al., 2006) as well as motion, not only of the head but also from out-of-field object including respiratory thoracic movement. To correct for these spurious signal changes and robustify registration, an intensity bias was fitted non-parametrically to each slice data, with higher weighting of white-matter where limited BOLD signal changes of interest were expected. This bias, aiming to model slice-specific acquisition perturbations, was fitted and removed prior to any interpolation that could mix signal and artifacts from different slices. Finally, kernel regression of resulting scattered data was performed at segmented and template registered gray-matter location to reconstruct BOLD signal in a space directly enabling between-subjects statistical analysis.

Evaluation of the method, by processing single-slice simulated data, showed that generated dynamic motion and intensity bias parameters is retrieved with low error ranging below two tenth of mm (see Figure 3) and below 4% of the multiplicative field respectively. In comparison, volume-wise motion registration provided less accurate motion estimates (see Figures 2, 3), as this approach was not able to track motion at sub-volume resolution nor was constrained for continuity, leading to systematically larger errors for both slow or rapid movements.

As illustrated in Figure S1, distortions are appropriately taken into account by the fieldmap which is a prerequisite to accurate anatomical localization of activity when interpolation of the gray-matter timecourse is used with limited smoothing. The reduction of measures of signal variability reflects the removal of sources of variance with high-frequency characteristics that are inconsistent with the hemodynamic processes. The large reduction of statistical dependency between motion parameters and signal indicates that accurate spatial registration decreases the motion related signal intensity changes and avoids introduction of large-scale spurious signal variations due to erroneous estimates.

One of the limitations of our algorithm is that its accuracy depends on the variable amount of brain coverage given by the slices which relates to available information for registration and intensity correction. The common use of axial slicing scheme provides caudal slices, covering brainstem and cerebellum, and rostral slices, covering cortex, with relatively little anatomical information, as compared to mid-brain, leading to instability of the registration and outliers. While the Kalman online filtering of motion estimates ensures that such error is limited by propagating information from previous slices, multiple successive outliers can still occur and affect the accuracy. For this reason, the algorithm was expected to perform better in acquisition sequences that provide continuous rich anatomical information, such as SMS, which slices, regularly spaced to limit spatio-temporal overlap, also ensures constant coverage of large neo-cortical areas with rich structural content. To counteract the error in case of low-informational content would be for the state space to model not only position but also it's first and second order derivatives, velocity and acceleration, which are expected to vary smoothly. As the first frame is used as the reference for registration, it is required to be motion-free, and a careful quality assessment is thus required to avoid slices to be registered to a corrupted volume. Future work should focus on registering directly to high-resolution anatomical image, which should increase the motion estimate quality.

The implemented bias correction suppresses low-frequency intensity changes as measured from the white matter regions, which however have been reported to show small signal changes correlated to behavioral task design. While this phenomenon have not been widely studied nor explained, the removal of task-relevant signal from white-matter could decrease the signal of interest in neighboring gray-matter regions. Nevertheless, the use of signal from white-matter as regressed confounds in resting-state fMRI study have been widely used, and our high-pass filtering only removes the large-scale intensity changes in the signal, preserving fine-grained activity patterns. The direct interpolation from raw data to the segmented cortical and sub-cortical gray-matter is performed using a Gaussian kernel regression with limited bandwidth, which optionally further restrict the bandwidth in the direction perpendicular to the cortical surface in order to reduce smearing across tissues and sulci. While this performs satisfactorily, future work should focus in the choice of kernel that extract fine-scale activity with reduced noise sensitivity, notably for laminar level analysis of brain activity at ultra-high-field MRI (Polimeni et al., 2010; Goense et al., 2012; Huber et al., 2017) for which surface-based preprocessing and analysis are particularly adapted. While the kernel weights prioritize local information but can interpolate signal from further data point in the case of locally sparser sampling, degenerate cases of large sampling gap induced by rapid large movement could yet result in missing data. Future work will be required to perform not only spatial but also temporal interpolation to avoid such problem while also allowing to correct for slice acquisition timing differences.

While other methods directly used four dimensional data simultaneously estimate motion and distortions in an iterative process (Andersson et al., 2017), we chose an online filtering framework in which data are processed sequentially, as data are acquired. The former could show improved performance benefiting from a global optimization criterion, while our method achieves lower computational cost and constant memory requirement. The computational complexity is linear with number of acquired volume, which accommodates processing of longer scans with higher temporal and spatial resolution, enabling as well online processing for real-time applications.

Static fieldmap based correction of distortion enables the removal of the bulk of anatomical inaccuracy of EPI image. However, in long scanning with motion and scanner drifts, the B0 field evolves over time. Moreover, with increasing resolution in phase encoding direction, fast cyclic B0 fluctuations generated essentially by respiratory motion are observable in serial EPI acquisition. Proper correction would require either a continuous measure of the B0 in-homogeneity changes that can be extracted from evolution of MRI phase information (Yeo et al., 2008) which is generally discarded, or retrieved with computational non-linear registration. The latter option could be applied after linear registration of each slice to further improve anatomical accuracy and thus removing spurious motion-induced signal changes.

The proposed method provides a framework in which other slice-specific artifacts correction, such as B1 unwarping or eddy currents, or analysis, such as fitting R2^*^ parameters from multi-echo EPI, could be conducted prior to between slice interpolation, better matching the sequential order of acquisition.

## Conclusion

We developed a novel method that retrospectively processes raw individual fMRI data into an analysis-ready group template space, by applying slice-level motion tracking with concurrent distortion and intensity correction. Experimental results on simulated data show that this technique successfully and robustly remove significant motion-induced confounds. Analysis of real fMRI data with the proposed integrated method, in comparison to conventional preprocessing, highlights its benefits in terms of artifacts correction, signal quality, and statistical significance. The increase in signal quality compared to mainstream volume-based correction is thought to generalize to other scanner models or acquisition sequences and particularly adapt to novel high-resolution multi-slice techniques used in cutting-edge research projects. These improvements also provide more stable patterns of activity facilitating investigation of cerebral information representation in healthy or clinical populations for which motion, by interacting with magnetic resonance physics, deteriorates fine-scale measures of brain activity.

## Author contributions

BP: conceptualized the method, developed the software, analyzed the data, and wrote the manuscript; BP, AB, JD, and HB: edited the manuscript.

### Conflict of interest statement

The authors declare that the research was conducted in the absence of any commercial or financial relationships that could be construed as a potential conflict of interest.
