# Supplementary material for: Integrated fMRI Preprocessing Framework Using Extended Kalman Filter for Estimation of Slice-Wise Motion
Source: Front Neurosci. 2018 Apr 26;12:268. doi: 10.3389/fnins.2018.00268 (PMC5932184; doi:10.3389/fnins.2018.00268)
Supplement: Supplementary file 1 [file Image_1.pdf]

Supplementary Material

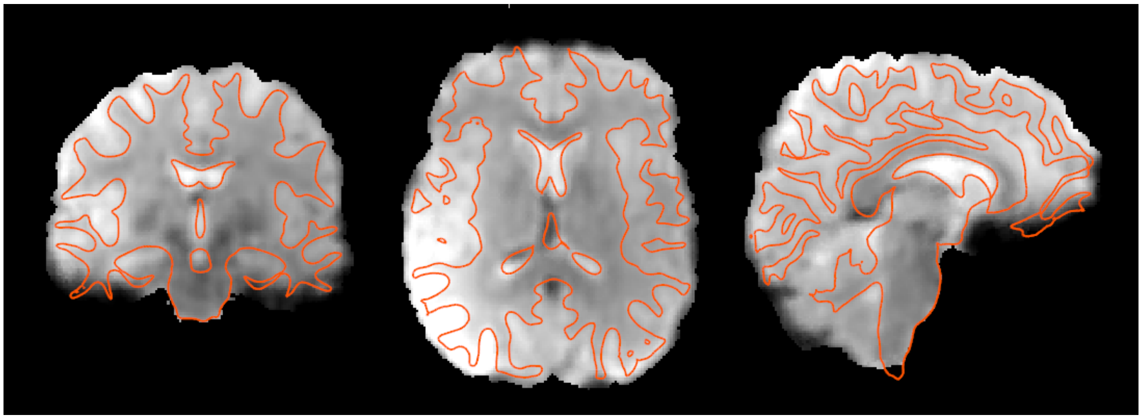

Figure 1: Interpolated volume overlaid with T1-based white-matter boundary to illustrate anatomical correspondence following distortion correction

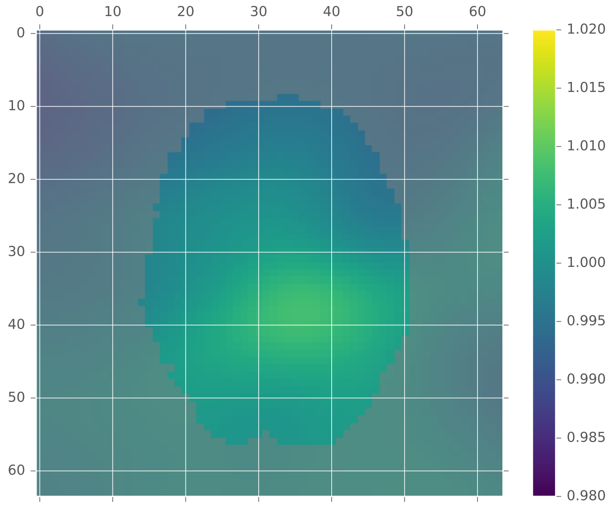

Figure 2: Example of slice bias estimated for a single slice on a real dataset, with brain mask overlaid

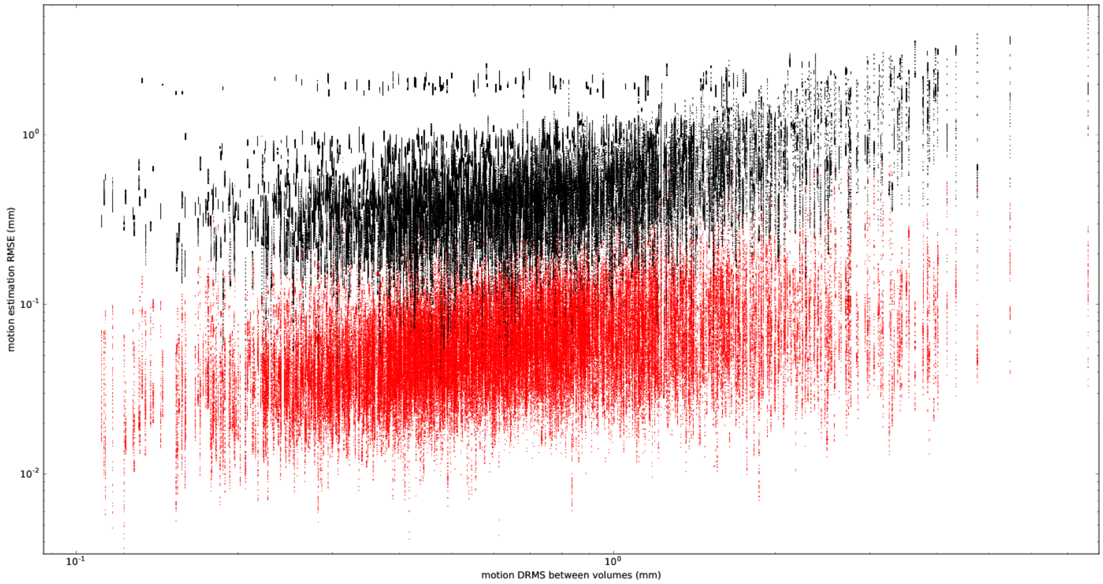

Figure 3: Log-log plot of the motion error by slice as a function of average volume displacement, for volumewise correction (black) and proposed slicewise correction (red), showing the latter benefit across movement amplitude.

A.1: Algorithm pseudo-code

inputs:

$Y =$ input_data

$\tau =$ registration to T1

$t = 0$

$Q = Id(6)\times$ transition_state_covariance

$x_0 = [0, 0, 0, 0, 0, 0]$

$P_0 = Id(6)\times$ init_state_covariance

for each volume v in Y:

    for each slice/slab data y in v:

        $t = t + 1$

        $x^0_{t|t-1} = Ax_{t-1|t-1}$

        $P^0_{t|t-1} = P_{t-1|t-1} + Q$

        until convergence or max iteration:

            $h, H =$ resample(ref,$x^i_{t|t}$)

            $m, e =$ resample_mask_and_pve(mask, pve, $\tau, x^i_{t|t}$)

            $b =$ compute bias(e, y, h)

            $K^i_t = P_{t|t-1}{H^i}^T[H^i P_{t|t-1}{H^i}^T + R]^{-1}$

            $x^{i+1}_{t|t} = x^i_{t|t-1} - K^i_t[\frac{y}{b} - h(x^i_{t|t})]$

        $P_{t|t} = [I - K^N_t H^N]P_{t|t-1}$

    scatter_data_resampling($x[(t-nslabs)..t], Y[(t-nslabs)..t(nslabs)]$)

A.2: Simulated movement generation code

```
import numpy as np
import dipy.align.transforms
def random_walk_motion_dipy(ntps,dt):
    # npts : number of time points
    # dt : spacing in seconds between time points
    states = np.zeros((ntps,6))
    motion = np.empty((ntps,4,4))
    t = dipy.align.transforms.RigidTransform3D()
    state, speed, acc_slow, acc_fast = np.zeros(6), np.zeros(6), np.zeros(6), np.zeros(6)
    for i in range(ntps):
        speed_norm = np.sqrt(np.square(speed).sum())
        # 5%  chance of fast acceleration changing
        if np.random.rand() > .95:
            # slight bias to return to center position
            acc_fast[:] = np.random.logistic(-state*.1,1e-1,6)
            # zeros some accceleration parameters to decorrelate the fast motion
            acc_fast *= np.random.uniform(size=6) > .6
        # 10% chance of slow acceleration changing
        if np.random.rand() > .9:
            acc_slow[:] = np.random.normal(0,5e-3,6)
        # chances of resetting acceleration increase with speed
        if np.random.rand() < dt*speed_norm:
            acc_fast[:] = 0
            speed *= 5e-2
        acc = (acc_slow+acc_fast)

        speed += acc
        state += speed*dt # scale by time resolution
        states[i] = state
        # rotation is transformed from "mm translation" to radians for a ~100mm head radius
        states[i,:3] *= 1e-2
        motion[i] = t.param_to_matrix(states[i])
    return motion, states
```
